# Incidence of immediate allergic reactions to mRNA COVID-19 vaccines in adults with drug allergies and other allergic disorders

**DOI:** 10.1097/MD.0000000000029571

**Published:** 2022-07-29

**Authors:** Ivan Marković, Marina Božan, Tomislav Perković, Katarina Paušek, Vanja Nedeljković, Marina Perković, Tomislav Kelava, Marinko Artuković, Asja Stipić Marković

**Affiliations:** a Special Hospital for Pulmonary Diseases, Zagreb, Croatia; b Department of Physiology and Immunology, University of Zagreb, School of Medicine, Zagreb, Croatia.

**Keywords:** anaphylaxis, COVID-19, drug hypersensitivity, incidence, mRNA vaccines

## Abstract

Concerns have been raised about allergic reactions to messenger ribonucleic acid (mRNA) coronavirus disease 2019 (COVID-19) vaccines. A history of allergic reactions, including anaphylaxis to drugs, has been frequently reported in individuals with anaphylaxis to mRNA vaccines.

To estimate the rate of immediate allergic reactions in patients with a history of drug allergy or other allergic disorders.

We included adult patients who had received at least 1 dose of an mRNA COVID-19 vaccine at the Special Hospital for Pulmonary Diseases between March 1, 2021, and October 1, 2021, and who reported a history of drug allergy or other allergic diseases (asthma, allergic rhinitis, atopic dermatitis, food or insect venom allergy, mastocytosis, idiopathic anaphylaxis, acute or chronic urticaria, and/or angioedema). Immediate allergic reactions, including anaphylaxis, occurring within 4 hours of vaccination were recorded.

Six immediate allergic reactions were noted in the cohort of 1679 patients (0.36%). One patient experienced anaphylaxis (0.06%), which resolved after epinephrine administration, and the other reactions were mild and easily treatable.

Most patients with a history of allergies can safely receive an mRNA COVID-19 vaccine, providing adequate observation periods and preparedness to recognize and treat anaphylaxis.

## 1. Introduction

Coronavirus disease 2019 (COVID-19) is caused by the novel coronavirus, severe acute respiratory syndrome coronavirus 2, which spread rapidly in 2020, causing a global pandemic with devastating medical and socioeconomic consequences. In December 2020, the US Food and Drug Administration issued an Emergency Use Authorization for Pfizer-BioNTech (BNT162b2) COVID-19 vaccine and Moderna COVID-19 vaccine (mRNA-1273), later named Comirnaty and Spikevax, respectively.^[1,2]^ Both messenger RNA (mRNA) vaccines were also approved by the European Medicines Agency by January 6, 2021.^[3,4]^

Systemic allergic reactions to vaccine components are generally rare, with the incidence of anaphylaxis estimated at 1.31 per 1 million doses administered.^[5]^ Shortly after the implementation of mass vaccination for COVID-19, reports of anaphylaxis after the first dose emerged, occurring at a rate of 11.1 cases per million doses administered of Pfizer-BioNTech vaccine,^[6]^ and 2.5 cases per million doses administered of Moderna COVID-19 vaccine.^[7]^ Furthermore, the rate of nonanaphylactic immediate allergic reactions to the first dose of Pfizer-BioNTech vaccine was estimated to be at least 4-fold, approaching 45.4 cases per million doses administered.^[6]^ Since these early reports, additional cases of anaphylaxis have been identified in the course of the massive vaccination programs. Based on information through January 18, 2021, the estimated reporting rates for anaphylaxis in the United States were 4.7 cases per million doses administered of Pfizer-BioNTech vaccine and 2.5 cases per million doses administered of Moderna COVID-19 vaccine. In most (89%) anaphylaxis cases, symptoms occurred within 30 minutes of vaccination, and most individuals with anaphylaxis had a history of allergy (77% of Pfizer-BioNTech vaccine recipients and 84% of Moderna vaccine recipients), including some with previous anaphylaxis.^[8]^ More recently, based on data from the US Vaccine Adverse Event Reporting System (VAERS) and the European EudraVigilance, the mean anaphylaxis rate was estimated to be 10.67 cases per million doses administered of COVID-19 vaccines. This rate was comparable to that of other common vaccines, such as tick-borne encephalitis, human papillomavirus, and herpes zoster vaccine. Overall, there were 0.06 fatal anaphylaxis cases per 1 million COVID-19 vaccine doses, according to VAERS and EudraVigilance.^[9]^

Allergic reactions to COVID-19 vaccines are thought to be driven by the excipients. Polyethylene glycol in mRNA vaccines and polysorbate 80 in viral vector-based vaccines are suspected as possible culprits, although this has not yet been proven.^[10]^ Non–immunogloblin E immunologic mechanisms, possibly complement activation-related pseudoallergy, are thought to play a role in allergic reactions to polyethylene glycol.^[11]^

A history of allergic reactions, including previous anaphylaxis to drugs, has been frequently reported in individuals with anaphylaxis to mRNA vaccines.^[6–8]^ However, the risk factors for immediate allergic reactions remain unclear. Data from VAERS indicated that the relative incidence of anaphylaxis to mRNA COVID-19 vaccines was twice as high in recipients with a history of allergy, and 7 times as high in recipients with a history of anaphylaxis.^[12]^

The objective of this study was to estimate the incidence of immediate hypersensitivity reactions, including anaphylaxis to mRNA COVID-19 vaccines, in patients with a history of allergic reactions to drugs or other allergic diseases.

## 2. Participants and Methods

The COVID-19 vaccine referral center was established at the Special Hospital for Pulmonary Diseases in Zagreb, Croatia, for the vaccination of individuals who were considered to be at increased risk for allergic reactions to the vaccine. Patients referred to the center included individuals aged 18 years or older with a history of drug allergy and/or other allergic disorders. These patients had often been denied receiving a COVID-19 vaccine at regular vaccination sites or at their primary care physician’s office because of the perceived risk of severe allergic reactions.

This cohort study followed the Strengthening the Reporting of Observational Studies in Epidemiology reporting guidelines for observational studies^[13]^ and it was conducted in accordance with the Declaration of Helsinki.^[14]^ The study was approved by the Institutional Review Board of the Special Hospital for Pulmonary Diseases. Written informed consent for publication was obtained from all patients.

Patients at “higher risk” for allergic reactions who received at least 1 dose of an mRNA COVID-19 vaccine (Comirnaty or Spikevax) at our center between March 1, 2021, and October 1, 2021, were included in the cohort. We defined “higher risk” as a history of drug allergy or other allergic disease (asthma, allergic rhinitis, atopic dermatitis, food allergy, insect venom allergy, mastocytosis, idiopathic anaphylaxis, acute or chronic urticaria, and/or angioedema). Patients referred for vaccination for other reasons who did not meet the definition of “higher risk” were excluded from the study.

Demographic data (age and sex) and medical history were obtained for each patient. Baseline blood pressure, heart rate, and peripheral oxygen saturation were recorded. Premedication with antihistamines was not recommended prior to vaccination, but patients taking antihistamines or glucocorticoids for treatment of an underlying chronic condition were advised to continue regular treatment. The minimum observation period after vaccine administration was 60 minutes after the first dose and 30 minutes after the second dose. During this period, adverse reactions and the medications administered to treat them were recorded. Before discharge, patients were instructed to contact our center or the nearest health care provider in case of any delayed adverse events.

The primary outcome was an immediate allergic reaction to the first or second dose of mRNA COVID-19 vaccine. For the purpose of this study, an immediate allergic reaction was defined as a reaction that occurred within 4 hours of vaccine administration, presented with compatible symptoms and/or signs, and was observed and/or treated by a healthcare professional. Self-reported allergic symptoms that occurred within 4 hours but were not observed or treated by a healthcare professional, or allergic symptoms that occurred after 4 hours were not analyzed further. To improve the diagnostic certainty, the medical records of all patients with suspected immediate allergic reactions were reviewed by a team of 3 allergy specialists. Adverse events that were classified as nonallergic were excluded from further analysis. Patients with symptoms and signs consistent with an immediate allergic reaction were followed up by an allergist, and a detailed description of the reaction was obtained. These patients were also offered skin testing (prick and intradermal testing) with the culprit vaccine.

The secondary outcome was a severe allergic reaction (anaphylaxis) to the mRNA COVID-19 vaccine. The modified World Allergy Organization (WAO) grading system was used to grade allergic reactions.^[15]^ The Brighton Collaboration case definition criteria for anaphylaxis were used to determine the level of diagnostic certainty.^[16]^

### 2.1. Statistical analysis

Data are presented as median with interquartile range (IQR) for nonnormally distributed numeric variables or as mean ± standard deviation for normally distributed numeric variables. Normality of distribution was tested using the Kolmogorov–Smirnov test; for a *P* value of <.05, a nonnormal distribution was accepted. Categorical data are presented as frequency counts and percentages. GraphPad Prism version 6 for Windows (GraphPad Software Inc., La Jolla, CA) was used for analysis.

## 3. Results

We identified 1741 individuals who received at least 1 dose of the mRNA COVID-19 vaccine at the COVID-19 vaccine referral center in the Special Hospital for Pulmonary Diseases between March 1, 2021, and October 1, 2021. Sixty-two patients were not included in the study because they did not meet our definition of “higher risk” for allergic reactions to the vaccine.

Of the 1679 vaccine recipients included in the cohort, 1211 (72.1%) were women and 468 (27.9%) were men. All subjects were Caucasian. The median (IQR) age of participants was 60 (47–69) years. Most participants (96.5%) received both doses of mRNA COVID-19 vaccine at our center. The majority (83.3%) received the BNT162b2 vaccine (Comirnaty).

A total of 1432 (85.3%) patients had a history of drug allergy, most of which were self-reported, and only a minority (5.5%) were confirmed by allergy or clinical pharmacology specialists. A total of 720 patients (42.9%) reported a history of allergic diseases other than drug allergies. Of these, allergic rhinitis (15.1%), food allergy (12.7%), asthma (11.1%), and insect venom allergy (9.7%) were the most common. Of note, 2 patients had systemic mastocytosis (Table 1).

**Table 1:** Demographic data and reported history of allergic disorders.

**Demographic data**	**n (%**)
Female	1211
Male	468
Total	1679
Age (yr), median (IQR)	60 (47–69)
History of allergic disorders	n (%)
Drug allergy	1432 (85.3)
Self-reported	1353 (94.5)
Confirmed by evaluation	79 (5.5)
Food allergy	213 (12.7)
Insect venom allergy	163 (9.7)
Asthma	186 (11.1)
Allergic rhinitis	253 (15.1)
Atopic dermatitis	13 (0.8)
Acute urticaria and/or angioedema	62 (3.7)
Chronic urticaria and/or angioedema	35 (2.1)
Mastocytosis	2 (0.1)
Idiopathic anaphylaxis	7 (0.4)

In this cohort, 22 immediate reactions were initially reported, which were labeled as allergies. After further evaluation by allergy specialists, 16 of these adverse events were considered to not represent hypersensitivity reactions and were excluded from further analysis. A total of 6 immediate allergic reactions were identified (0.36%). Five of these were mild (WAO grade 1–2) and resolved soon after treatment with antihistamines and/or glucocorticoids. All reactions occurred within 30 minutes after vaccination. Only 1 patient experienced anaphylaxis (0.06%) with respiratory and gastrointestinal symptoms (WAO grade 3, Brighton level 3 of diagnostic certainty). The event occurred 20 minutes after administration of the first dose of Comirnaty vaccine. The patient was treated with intramuscular epinephrine (0.5 mg) followed by prompt resolution of all symptoms. Of the 16 patients who experienced a nonallergic immediate adverse event, 2 had vasovagal syncope, and others presented with pruritus without rash, isolated nausea, lip tingling, or globus sensation.

In the subgroup of 6 patients who experienced an immediate allergic reaction, all were female, with a median age (IQR) of 60 (53–66) years. Five patients received the Comirnaty vaccine (lot numbers EX3599, FD4500, FE6208 and FE6029) and 1 patient received the Spikevax vaccine (lot number 3001659). All patients reported a history of drug allergy, and half of them, including the patient with anaphylaxis, reported a history of allergy to multiple drugs. In 4 patients the reaction occurred after the first dose of vaccine and in 2 patients after the second dose (Table 2).

**Table 2:** Allergic reactions to mRNA COVID-19 vaccines.

**Patient no.**	**Age, yr**	**Sex**	**History of drug allergies**	**History of other allergic disorders**	**Vaccine**	**Dose**	**Symptoms**	**WAO grade**	**ID test to vaccine**	**Further dose of COVID-19 vaccine**
1	66	F	Piperacillin+tazobactam, ertapenem, cephalosporins	None	Spikevax	2	Angioedema	1	Positive	Received and tolerated booster dose of a viral vector-based vaccine
2	50	F	Penicillin, paracetamol, ASA, TMP/SMX	Acute urticaria	Comirnaty	1	Angioedema and erythema	1	Not done	Not received
3	58	F	ASA	Food allergy	Comirnaty	1	Angioedema	1	Positive	Not received
4	62	F	Iodinated contrast agent	None	Comirnaty	1	Metallic taste, nausea, angioedema	2	Negative	Received and tolerated second dose of Comirnaty vaccine
5	53	F	Azithromycin, erythromycin, clindamycin, ASA, bromhexine	None	Comirnaty	1	Epiphora, rhinorrhea, nasal congestion, pruritus, dyspnea, chest tightness, nausea and vomiting	3	Negative	Not received
6	69	F	Penicillin	Allergic rhinitis	Comirnaty	2	Urticaria	1	Not done	Not received

Skin testing with the causative vaccine was performed in 4 patients (prick test with undiluted vaccine followed by intradermal test with 1:100 and 1:10 dilutions). The prick test was negative in all patients, and the intradermal test was positive in 2 patients. Notably, in the patient with anaphylaxis, skin testing with Comirnaty vaccine was performed 4 weeks after the reaction, and the intradermal test was negative. Of the 4 patients who reacted to the first dose, only 1 patient who had a negative intradermal test received and tolerated the second dose of the same vaccine. The remaining 3 patients, including the patient with anaphylaxis, chose not to receive further doses. One patient who had experienced an allergic reaction to only the second dose of mRNA vaccine (Spikevax) and had a positive intradermal test to the causative vaccine received a booster dose of the viral vector vaccine (COVID-19 Vaccine Janssen), which she tolerated well.

Of note, 2 patients with systemic mastocytosis, 7 patients with anaphylaxis due to an unknown trigger, and 1 patient with a history of an immediate allergic reaction to a laxative containing macrogol 3350 all tolerated mRNA COVID-19 vaccine.

## 4. Discussion

Concerns have been raised about the safety of mRNA COVID-19 vaccines, particularly with regard to severe allergic reactions, leading to increased vaccine hesitancy and impediments to mass vaccination programs. Anaphylaxis to an mRNA COVID-19 vaccine remains a rare event, with an estimated mean rate of 0.001%.^[9]^ Nonanaphylactic immediate allergic reactions have occurred more frequently, with the highest reported rate reaching 2.1% in a population of US health care employees.^[17]^

However, vaccine adverse reactions are generally easily misinterpreted as allergic, especially in the absence of objective signs of an allergic reaction, when only subjective symptoms are present. Therefore, the reported rates of immediate allergic reactions to COVID-19 vaccines may have been overestimated as a consequence of ascertainment bias.^[18]^ Evaluation of these reactions by an allergy specialist may significantly improve the level of diagnostic certainty. In our study, only immediate reactions directly observed by medical personnel were included in further analysis, while self-reported symptoms after discharge were not considered. After a thorough evaluation by a team of allergy specialists, a total of 16 of 22 immediate reactions believed to be allergic reactions were classified as nonallergic because of the absence of compatible symptoms and/or objective signs, regardless of whether patients received treatment for an allergic reaction.

A significant proportion of patients who experienced allergic reactions to mRNA COVID-19 vaccines reported previous allergic reactions, including anaphylaxis to various drugs and/or other allergens.^[8]^ Prior anaphylaxis has been reported in around a third of patients with anaphylaxis to the vaccine, and a history of allergies or prior allergic reactions of any intensity in up to 77% of them.^[8,17]^ According to Macy et al,^[19]^ patients with acute-onset hypersensitivity reactions to mRNA COVID-19 vaccines were more likely to be female, to report intolerance to multiple drugs, and to carry diagnoses of chronic urticaria, asthma, food allergy and anxiety. Li et al^[20]^ indicated that individuals with a self-reported history of high-risk allergy, defined as any history of a severe allergic reaction to an injectable drug, vaccine, or other allergen, were approximately 2.5 times more likely to report an allergic reaction to the mRNA COVID-19 vaccine, with the risk being highest for hives and angioedema.

In our allergic cohort, the rate of immediate allergic reactions to mRNA COVID-19 vaccines was 0.36%, and the rate of anaphylaxis was 0.06%. These rates were higher than those reported in an early report from VAERS and in another study that included subjects who were not selected based on history of allergy (0.006% and 0.028% for immediate hypersensitivity reactions irrespective of severity; 0.001% and 0.0003% for anaphylaxis).^[6,19]^ In contrast to our study, these 2 reports included data from electronic health records that were retrospectively analyzed, so the results may still have been overestimated due to the inherent propensity for ascertainment bias.

To our knowledge, this is the second study to prospectively investigate the rate of immediate allergic reactions after administration of an mRNA COVID-19 vaccine in a selected population with allergic disorders. Shavit et al investigated the rate of allergic reactions to mRNA COVID-19 vaccines in a cohort of 429 Israeli patients classified as “highly allergic.” The rate of mild allergic reactions was 1.4% and the rate of anaphylaxis was 0.7%.^[21]^ These rates were considerably higher than in our patients. However, this “highly allergic” group was selected on the basis of a set of more stringent criteria than our group, which included prior anaphylaxis to drugs or vaccines, multiple drug allergies, multiple allergies, or mast cell disorders. In this cohort, prior anaphylaxis was reported in 63.2%.

A limitation of this study was the use of self-reported data on drug allergies. Only a minority of participants in our cohort had been previously evaluated by an allergy or clinical pharmacology specialist to confirm or exclude hypersensitivity to drugs. Therefore, a certain proportion of patients may have had adverse events of different types and/or mechanisms mislabeled as drug allergies in their medical records. The same may be true for some food allergies. Nevertheless, although it was virtually impossible to ascertain the exact proportion of patients mislabeled as drug-allergic, the patients in our cohort were generally those whose vaccination was deferred by their physicians or vaccination teams due to fear of severe allergic reactions and who were referred to our center for vaccination in a special setting.

In addition, the number of identified allergic reactions in our cohort was small, and therefore no conclusions about risk factors could be drawn. Further studies with a larger cohort and a higher number of immediate allergic reactions are needed.

In conclusion, allergic reactions to mRNA COVID-19 vaccines in patients with a history of drug allergy and other allergic diseases are rare and most are mild and easily treatable. Most of these patients can safely receive a vaccine in a regular setting. However, adequate observation time of at least 30 minutes after vaccination should be ensured, and health care professionals should be prepared to recognize and treat anaphylaxis.

## Author contributions

Conceptualization: Ivan Marković, Asja Stipić Marković

Data curation: Tomislav Perković, Katarina Paušek

Formal analysis: Tomislav Kelava

Investigation: Tomislav Perković, Marina Božan

Methodology: Ivan Marković, Marina Perković

Project administration: Marinko Artuković

Resources: Vanja Nedeljković

Supervision: Asja Stipić Marković

Visualization: Vanja Nedeljković

Writing – original draft: Ivan Marković, Marina Božan

Writing – review & editing: Tomislav Kelava, Marina Perković
